# Textbook outcome and survival of robotic versus laparoscopic total gastrectomy for gastric cancer: a propensity score matched cohort study

**DOI:** 10.1038/s41598-021-95017-3

**Published:** 2021-07-28

**Authors:** Chul Kyu Roh, Soomin Lee, Sang-Yong Son, Hoon Hur, Sang-Uk Han

**Affiliations:** 1 Department of Surgery, Ajou University School of Medicine, 164, World cup-ro, Yeongtong-gu, Suwon-si, Gyunggi-do 16499 Republic of Korea; 2 Gastric Cancer Center, Ajou University Medical Center, Suwon, Republic of Korea

**Keywords:** Gastrointestinal cancer, Gastroenterology, Oncology

## Abstract

Textbook outcome is a composite quality measurement of short-term outcomes for evaluating complex surgical procedures. We compared textbook outcome and survival of robotic total gastrectomy (RTG) with those of laparoscopic total gastrectomy (LTG). We retrospectively reviewed 395 patients (RTG, n = 74; LTG, n = 321) who underwent curative total gastrectomy for gastric cancer via minimally invasive approaches from 2009 to 2018. We performed propensity score matched analysis to adjust for potential selection bias. Textbook outcome included a negative resection margin, no intraoperative complication, retrieved lymph nodes > 15, no severe complication, no reintervention, no unplanned intensive care unit admission, hospitalization ≤ 21 days, no readmission after discharge, and no postoperative mortality. Survival outcomes included 3-year overall and relapse-free survival rates. After matching, 74 patients in each group were selected. Textbook outcome was similar in the RTG and LTG groups (70.3% and 75.7%, respectively), although RTG required a longer operative time. The quality metric least often achieved was the presence of severe complications in both groups (77.0% in both groups). There were no differences in the 3-year overall survival rate (98.6% and 89.7%, respectively; log-rank P = 0.144) and relapse-free survival rate between the RTG and LTG groups (97.3% and 87.0%, respectively; log-rank P = 0.167). Textbook outcome and survival outcome of RTG were similar to those of LTG for gastric cancer.

## Introduction

Quality assurance in surgical oncology is an important factor in evaluating the outcomes of surgical procedures^1,2^. Traditionally, quality assessment for gastrectomy has focused on surgical mortality and morbidity^3–5^. Since it only provides information on single-quality indicators, it does not fully measure the complex aspects of perioperative surgical care in gastric cancer.

Investigators have been increasingly searching for standards for perioperative outcomes. In 2017, the Dutch Upper Gastrointestinal Cancer Audit group developed a composite measure defined as textbook outcome that combined 10 outcome parameters to assess the quality of surgical care for gastric cancer^6^. Several studies have reported that textbook outcome can be used as the standard for optimization of care and is associated with long-term survival in patients with gastric cancer^7–10^. Additionally, textbook outcome has been applied to comparisons of quality across surgical practices and institutions in various fields, such as colon, esophagus, liver, and pancreatic cancers^7,11–13^.

Recently, robotic gastrectomy showed reliable surgical outcomes as an alternative for laparoscopic gastrectomy in minimally invasive surgery for the treatment of gastric cancer^14–19^. However, these studies focused on single-quality indicators such as bleeding, retrieved lymph nodes, complications, and mortality to compare short-term outcomes. Unfortunately, no studies have yet utilized textbook outcome to compare short-term surgical outcomes between robotic and laparoscopic gastrectomy. Further, there have been few studies that compare survival outcomes of robotic total gastrectomy (RTG) with those of laparoscopic total gastrectomy (LTG).

Therefore, we aimed to compare the perioperative (based on textbook outcome) and survival outcomes between RTG and LTG for gastric cancer treatment.

## Methods

### Patients

We reviewed the prospectively collected data of patients who underwent minimally invasive (robotic or laparoscopic) total gastrectomy for gastric cancer from January 2009 to December 2018 at the Department of Surgery, Ajou University School of Medicine. All gastrectomy cases were performed by three surgeons (HSU, HH, and SSY). The exclusion criteria were as follows: palliative gastrectomy, emergency surgery, and remnant gastric cancer that required completion total gastrectomy. Patients were given the option of undergoing robotic or laparoscopic gastrectomy and each patient selected the type of surgery. Written informed consent was provided prior to surgery. The institutional review board of Ajou University Hospital, Suwon, Korea approved this study (approval number: AJIRB-MED-MDB-19-144).

### Surgical procedures

#### Robotic total gastrectomy

RTG was first initiated at our institution in January 2009. We performed RTG using the da Vinci^®^ S, Si, or Xi Systems (Intuitive Surgical, Sunnyvale, CA, USA). Typical surgical procedures for RTG have been previously described in detail^20^. We performed D1 + or D2 lymph node dissection (LND) based on the Korean and Japanese gastric cancer treatment guidelines^21,22^. Esophagojejunostomy was performed extracorporeally using circular staplers, or intracorporeally using linear staplers or by suture technique. Until 2014, a mini laparotomy was created in the upper midline (approximately 7 cm) after esophageal mobilization. Esophagojejunostomy was performed using a 25-mm or 21-mm circular stapler, and jejunojejunostomy was performed. Since 2015, esophagojejunostomy and jejunojejunostomy were performed intracorporeally using linear staplers. A 45-mm linear stapler was inserted individually between the esophagus and prepared Roux limb, and fired to form a side-to-side esophagojejunostomy. Suture technique involved an intracorporeally robot-sewn esophagojejunostomy. After transection of the esophagus, seromuscular sutures were performed to attach the esophageal stump and jejunum together.

#### Laparoscopic total gastrectomy

We performed LTG with lymphadenectomy based on standardized operative strategies, as described previously^20^. The extent of LND was decided according to the Korean and Japanese gastric cancer treatment guidelines^21,22^. The reconstruction method was performed by extracorporeal or intracorporeal anastomosis as completed in RTG.

### Textbook outcome

Textbook outcome is a composite of nine components involved in the perioperative process, including the oncologic resection, postoperative care, and discharge of gastric cancer patients. We excluded the intent of surgery, which was one of the parameters of textbook outcome, from the definition to reduce selection bias for survival analysis in this study.

Textbook outcome was defined as the percentage of patients who had (a) no intraoperative complication (intraoperative complication was defined as any deviation from the ideal intraoperative course, such as intraoperative transfusion, unintended adjacent organ injury or resection, and conversion from minimally invasive to open surgery for any reason), (b) tumor-negative resection margins (as defined in the final surgical pathology report), (c) > 15 lymph nodes retrieved, (d) no severe postoperative complication (severe complications were defined as grade II or more complications according to the Clavien–Dindo classification), (e) no re-intervention (endoscopic, radiological or surgical re-intervention was not considered), (f) no unplanned admission to the intensive care unit, (g) hospitalisation period of ≤ 21 days, (h) no postoperative mortality within 30 days after surgery, and (i) no readmission after discharge (readmission was defined as admission to the hospital within 30 days after discharge from the initial admission or an emergency department visit within 30 days after discharge). Textbook outcome was achieved when all these metrics were met.

### Follow-up

Follow-up data after surgery of all patients were collected from our institution’s database. Patients with stage I cancer were regularly surveyed according to standard protocol (i.e., at 3-month intervals for the first year, 6-month intervals for the next 4 years, and yearly thereafter). Patients with stage II cancer or higher were surveyed every 3 months for the first 2 years, every 6 months for the next 3 years, and yearly thereafter. Physical examination, laboratory testing, esophagogastroduodenoscopy, and imaging (ultrasonography or computed tomography) were conducted during the follow-up. Recurrence was assessed by radiology, endoscopy, surgery, or clinical signs of the disease.

5-Fluorouracil-based adjuvant chemotherapy (e.g., S-1 monotherapy or oxaliplatin with capecitabine) was suggested to patients with pathological stage II or III cancer. Survival status for all patients was confirmed. All-cause and cancer mortality data were acquired from the Korean National Cancer Registry. Patient follow-up was completed until death or the last follow-up for all patients (data cut-off: December 31, 2019).

### Propensity score matching

Propensity score matching analysis was performed to adjust for potential selection bias between the groups. Among clinicopathological variables, the covariates with a standardized mean difference (SMD) > 0.1 in the entire cohort were selected for matching to balance significant differences in baseline characteristics as follows: age, sex, body mass index (BMI), the American Society of Anesthesiologist (ASA) physical status, tumor size, and the pathological stage of gastric cancer. TNM stage was based on the 8th edition of the American Joint Committee on Cancer staging for gastric cancer. Individual propensity scores were calculated using a logistic regression model, and patients between the two groups were matched using the nearest-neighbor matching algorithm (ratio = 1:1 without replacement) with a caliper width of 0.25 standard deviation of the propensity score.

### Statistical analyses

Data analyses were performed using IBM SPSS Statistics for Windows, version 25.0 (IBM Corp., Armonk, NY, USA). Continuous variables were analyzed using the Student’s t*-*test to compare mean values. Categorical variables, which are presented as numbers (n) and percentages (%), were analyzed using the chi-square test or Fisher’s exact test. Overall survival was defined as the time of gastrectomy to the time of all-cause death. Relapse-free survival was defined as the time of gastrectomy to the time of tumor recurrence or all-cause death. Survival curves were estimated using the Kaplan–Meier method for the entire cohort and for the matched cohorts. To investigate prognostic variables associated with overall and relapse-free survival, Cox proportional hazard regression model was used for univariate and multivariate analysis. Statistical significance was defined as a *P* < 0.05.

### Ethics declarations

The study was conducted in accordance with the “Declaration of Helsinki”, and was approved by the institutional review board of Ajou University Hospital, Suwon, Korea (AJIRB-MED-MDB-19-144).

## Results

### Study cohort

Four hundred and thirty-four patients who underwent gastrectomy from 2009 to 2018 for gastric cancer by either robot or laparoscopy were identified. Of these, 39 patients were excluded who underwent palliative gastrectomy (n = 28) or completion total gastrectomy for remnant gastric cancer (n = 10), or emergency surgery due to cancer bleeding (n = 1). Finally, 395 patients (RTG, n = 74; LTG, n = 321) were included. After matching, 148 patients (74 patients per group) were analyzed to compare textbook and survival outcomes between the two groups (Supplementary Fig. S1).

### Patient characteristics

Before matching, the mean age of the RTG group (53.8 ± 11.6 years) was lower than that of the LTG group (60.7 ± 12.6 years; *P* < 0.001, SMD = 0.590). The RTG group (43.2%) exhibited a higher proportion of female sex than the LTG group (30.5%; *P* = 0.040, SMD = 0.255). The proportion of patients with ASA class I was higher in the RTG group than in the LTG group (67.6% versus [vs.] 47.0%; *P* = 0.007, SMD = 0.380). After propensity score matching, clinicopathological variables were well balanced within the SMD of 0.1 between the two groups (Table 1).

**Table 1:** Clinicopathologic characteristics before and after propensity score matching.

	Entire cohort			SMD	Matched cohort			SMD
	Robot (n = 74)	Laparoscopy (n = 321)	*P*		Robot (n = 74)	Laparoscopy (n = 74)	*P*	
Age, years	53.8 ± 11.6	60.7 ± 12.6	** < 0.001**	0.590	53.8 ± 11.6	54.6 ± 12.7	0.690	0.069
**Sex**			**0.040**	0.255			> 0.999	< 0.001
Male	42 (56.8)	223 (69.5)			42 (56.8)	42 (56.8)		
Female	32 (43.2)	98 (30.5)			32 (43.2)	32 (43.2)		
Body mass index, kg/m^2^	23.6 ± 2.9	24.0 ± 3.2	0.262	0.154	23.6 ± 2.9	23.8 ± 3.4	0.692	0.071
**ASA group**			**0.007**	0.380			> 0.999	0.051
I	50 (67.6)	151 (47.0)			50 (67.6)	51 (68.9)		
II	22 (29.7)	162 (50.5)			22 (29.7)	22 (29.7)		
III	2 (2.7)	8 (2.5)			2 (2.7)	1 (1.4)		
**Tumor histology**			0.238	0.160			> 0.999	0.027
Differentiated	39 (52.7)	195 (60.7)			39 (52.7)	38 (51.4)		
Undifferentiated	35 (47.3)	126 (39.3)			35 (47.3)	36 (48.6)		
**Tumor location**			> 0.999	0.072			0.723	0.116
Upper third	51 (68.9)	221 (68.9)			51 (68.9)	47 (63.5)		
Middle to lower third	23 (31.1)	100 (32.2)			23 (31.1)	27 (36.5)		
**Clinical T classification**			1.000	0.116			0.869	0.054
T1	39 (52.7)	167 (52.0)			39 (52.7)	37 (50.0)		
≥ T2	35 (47.3)	154 (48.0)			35 (47.3)	37 (50.0)		
**Clinical N classification**			1.000	0.057			> 0.999	0.029
N0	50 (67.6)	216 (67.3)			50 (67.6)	49 (66.2)		
≥ N1	24 (32.4)	105 (32.7)			24 (32.4)	25 (33.8)		
Tumor size, cm	3.5 ± 1.7	4.0 ± 2.9	0.078	0.294	3.5 ± 1.7	3.5 ± 2.6	0.982	0.005
**Pathological T classification**			0.813	0.053			0.314	0.013
T1	43 (58.1)	184 (57.3)			43 (58.1)	43 (58.1)		
T2	12 (16.2)	41 (12.8)			12 (16.2)	9 (12.2)		
T3	12 (16.2)	64 (19.9)			12 (16.2)	19 (25.7)		
T4	7 (9.5)	32 (10.0)			7 (9.5)	3 (4.1)		
**Pathological N classification**			0.592	0.134			0.382	0.091
N0	53 (71.6)	215 (67.0)			53 (71.6)	46 (62.2)		
N1	9 (12.2)	45 (14.0)			9 (12.2)	17 (23.0)		
N2	8 (10.8)	29 (9.0)			8 (10.8)	7 (9.5)		
N3	4 (5.4)	32 (10.0)			4 (5.4)	4 (5.4)		
Pathological TNM classification*			0.320	0.145			0.829	0.058
I	50 (67.6)	207 (64.5)			50 (67.6)	47 (63.5)		
II	15 (20.3)	52 (16.2)			15 (20.3)	18 (24.3)		
III	9 (12.2)	62 (19.3)			9 (12.2)	9 (12.2)		
**Adjuvant chemotherapy**^†^			> 0.999	0.065			> 0.999	0.086
Yes	22 (91.7)	105 (92.1)			22 (91.7)	25 (92.6)		
No	2 (8.3)	9 (7.9)			2 (8.3)	2 (7.4)		
**Chronological distribution**			0.115	0.205			0.869	0.054
2009–2013	36 (48.6)	123 (38.3)			36 (48.6)	34 (45.9)		
2014–2018	38 (51.4)	198 (61.7)			38 (51.4)	40 (54.1)		

Statistically significant values (*P* < 0.05) are given in bold.

Data are expressed as mean ± standard deviations or as n (%) unless otherwise specified.

*SMD* standardized mean difference, *ASA* American Society of Anaesthesiologists.

*According to American Joint Committee on Cancer 8th edition.

^†^For patients with Stage II or III.

### Operative outcomes

In the matched cohort, a longer operative time was required in the RTG group than in the LTG group (226.0 ± 56.9 vs. 193.2 ± 46.8 min; *P* < 0.001, SMD = 0.578). The estimated blood loss tended to be less in the RTG group than in the LTG group, although this was not significantly different. The esophagojejunostomy method was comparable between the groups. No conversion to laparoscopic or open surgery occurred in the RTG group; however, at any time during the operation, the surgeon had the option of conversion to conventional laparoscopy or open surgery for patients’ safety. Perioperative outcomes regarding the extent of dissection, number of retrieved lymph nodes, tumor-free resection margins, time to soft diet, and duration of hospital stay were comparable in both groups. The incidence and grade of complications were also similar in both groups. We did not experience any mortality within the first 30 days after total gastrectomy by either robot or laparoscopy (Table 2).

**Table 2:** Operative outcomes before and after propensity score matching.

	Entire cohort			SMD	Matched cohort			SMD
	Robot (n = 74)	Laparoscopy (n = 321)	*P*		Robot (n = 74)	Laparoscopy (n = 74)	*P*	
**Extent of dissection**			0.519	0.092			0.869	0.054
< D2	36 (48.6)	171 (53.3)			36 (48.6)	34 (45.9)		
≥ D2	38 (51.4)	150 (46.7)			38 (51.4)	40 (54.1)		
**Esophagojejunostomy method**			**0.010**	0.084			0.283	0.164
Circular stapler	37 (50.0)	163 (50.8)			37 (50.0)	41 (55.4)		
Linear stapler	34 (45.9)	158 (49.2)			34 (45.9)	33 (44.6)		
Suture technique	3 (4.1)	0 (0)			3 (4.1)	0 (0)		
Combined resection	5 (6.8)	28 (8.7)	0.651	0.078	5 (6.8)	4 (5.4)	> 0.999	0.053
Conversion*	0 (0)	4 (1.2)	0.595	NA	0 (0)	1 (1.4)	> 0.999	NA
Operation time, min	226.0 ± 56.9	191.7 ± 48.5	** < 0.001**	0.604	226.0 ± 56.9	193.2 ± 46.8	** < 0.001**	0.578
Estimated blood loss, ml	137.9 ± 155.5	153.6 ± 150.0	0.420	0.101	137.9 ± 155.5	149.1 ± 128.3	0.632	0.072
Proximal margin, cm	2.5 ± 1.5	2.8 ± 2.3	0.278	0.158	2.5 ± 1.5	2.8 ± 2.0	0.341	0.183
Distal margin, cm	11.9 ± 3.4	11.7 ± 4.2	0.644	0.063	11.9 ± 3.4	11.9 ± 4.0	0.989	0.002
Retrieved LNs, n	43.1 ± 14.9	42.9 ± 15.0	0.916	0.014	43.1 ± 14.9	46.0 ± 14.4	0.232	0.194
Time to start soft diet, days	6.1 ± 4.0	5.7 ± 2.8	0.377	0.109	6.1 ± 4.0	6.4 ± 4.8	0.670	0.078
Postoperative hospital stay, days	9.0 ± 5.0	8.2 ± 4.4	0.214	0.146	9.0 ± 5.0	9.2 ± 5.7	0.795	0.046
**Complications**			0.304	0.132			> 0.999	< 0.001
No	51 (68.9)	241 (75.1)			51 (68.9)	51 (68.9)		
Yes	23 (31.1)	80 (24.9)			23 (31.1)	23 (31.1)		
**Clavien–Dindo classification**			0.766	0.107			0.893	0.048
Grade I	6 (8.1)	20 (6.2)			6 (8.1)	6 (8.1)		
Grade II	9 (12.2)	29 (9.0)			9 (12.2)	6 (8.1)		
Grade III	6 (8.1)	24 (7.5)			6 (8.1)	8 (10.8)		
Grade IV	2 (2.7)	7 (2.2)			2 (2.7)	3 (4.1)		
Postoperative mortality	0 (0)	0 (0)	NA		0 (0)	0 (0)	NA	
Recurrence and/or death	6 (8.1)	53 (16.5)	0.072	0.306	6 (8.1)	11 (14.9)	0.303	0.246

Statistically significant values (*P* < 0.05) are given in bold.

Data are expressed as means ± standard deviations or as n (%) unless otherwise specified.

*SMD* standardized mean difference, *LNs* lymph nodes, *NA* not applicable.

*Conversion to laparoscopic or open surgery in the robotic group and conversion to open surgery in the laparoscopic group for any reason.

The detailed complications are listed in Supplementary Table S1. The early and late complication rates were comparable between the groups. In early complications, local and systemic complication rates were also similar. Leakages after esophagojejunostomy were identified in three patients after robotic gastrectomy (4.1%) and in seven patients after laparoscopic gastrectomy (2.2%). The patients with esophagojejunostomy leakage underwent endoscopic and radiological interventions or reoperation.

### Textbook outcome

**Figure 1:**
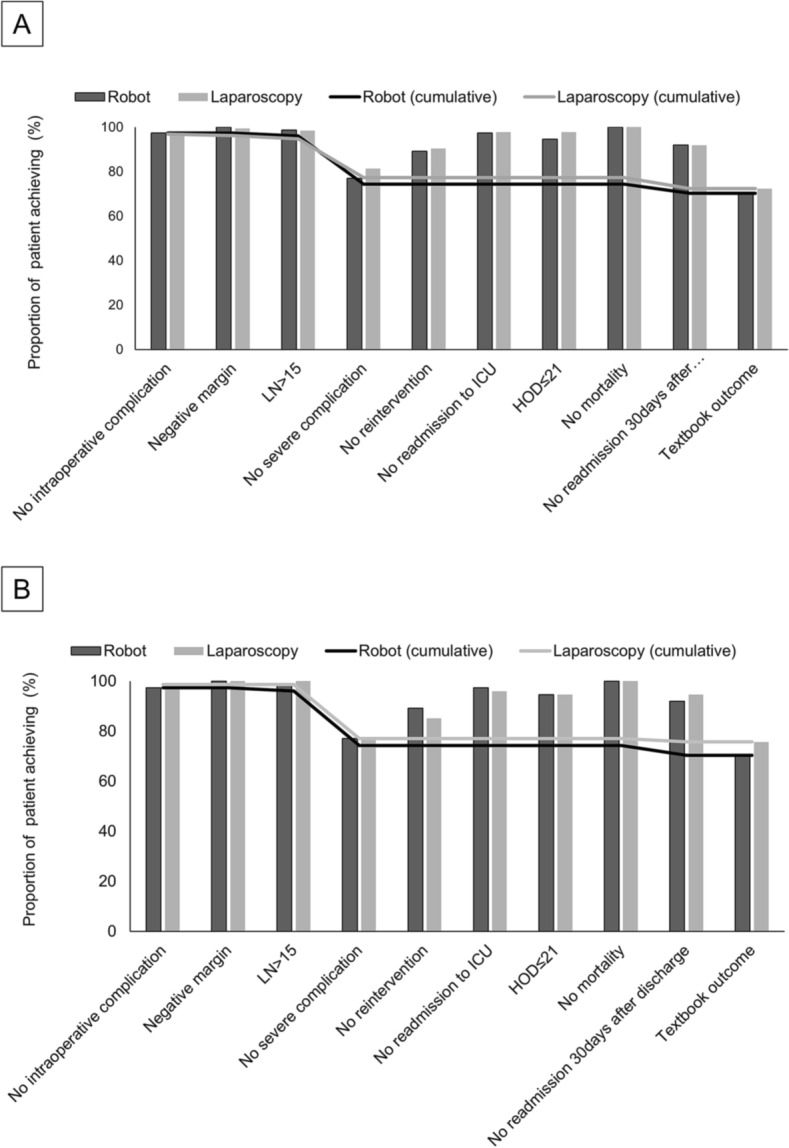
Proportion of patients achieving textbook outcome and each quality metric. (**A**) Entire cohort, (**B**) Matched cohort. *LN* lymph node, *ICU* intensive care unit, *HOD* hospital days.

Textbook outcomes were achieved in 70.3% (50 of 74) in the RTG group and in 75.7% (56 of 74) in the LTG group, which was not significantly different (*P* = 0.579). The distribution of each textbook outcome metric for both groups is presented in Fig. 1. Each quality metric was comparable between the two groups before and after matching. The quality metric that had the most negative impact on the proportion achieving textbook outcome was the absence of severe complications, which was achieved in only 77.0% in both groups (Supplementary Table S2). Detailed intraoperative complications after RTG and LTG are listed in Supplementary Table S3.

### Survival outcomes

**Figure 2:**
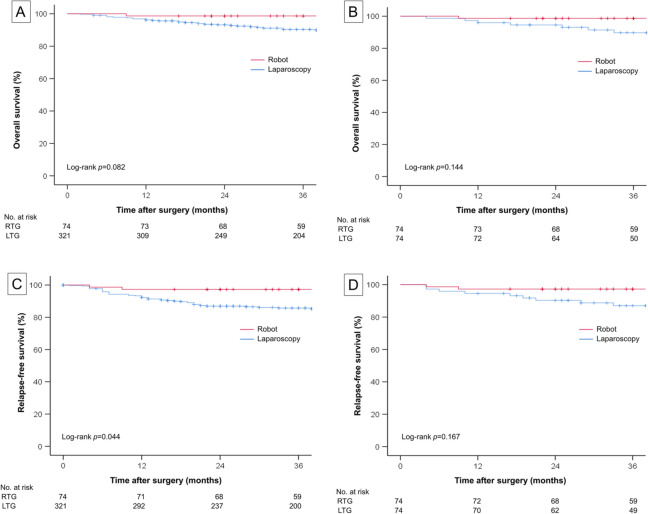
Kaplan–Meier curves for 3-year overall and relapse-free survival. (**A**) 3-year overall survival of the entire cohort. (**B**) 3-year overall survival of the matched cohort. (**C**) 3-year relapse-free survival of the entire cohort. (**D**) 3-year relapse-free survival of the matched cohort. *RTG* robotic total gastrectomy, *LTG* laparoscopic total gastrectomy.

The median follow-up periods for the entire cohort, RTG group, and LTG group were 51 (interquartile range 26–83 months), 60 (interquartile range 36–92 months), and 49 months (interquartile range 25–80 months), respectively. Five (6.8%) patients died in the RTG group, while 42 (13.1%) patients died in the LTG group. In the entire cohort, there was no difference in the 3-year overall survival rate between the RTG and LTG groups (98.6% and 90.3%, respectively; log-rank *P* = 0.082; Fig. 2A). After matching, the three-year overall survival rate also did not differ between the RTG and LTG groups (98.6 and 89.7%, respectively; log-rank *P* = 0.144; Fig. 2B). The numbers of deaths or recurrences in the RTG and LTG groups were six (8.1%) and 53 (16.5%), respectively. In the entire cohort, the 3-year relapse-free survival rate was higher in the RTG group (97.3%) than in the LTG group (85.7%, log-rank *P* = 0.044; Fig. 2C). However, after matching, 3-year relapse-free survival rate was comparable between the RTG (97.3%) and LTG (87.0%) groups (log-rank *P* = 0.167; Fig. 2D). Recurrence was recorded in two (2.7%) patients in the RTG group and in 31 (9.7%) patients in the LTG group; this difference was not statistically significant before and after matching (*P* = 0.061 and *P* = 0.442, respectively). Detailed recurrence patterns after RTG and LTG are listed in Supplementary Table S4.

### Prognostic factors for overall and relapse-free survival

In univariate analysis, the prognostic factors associated with overall survival were age, BMI, the ASA class, tumor size, estimated blood loss, pathological TNM stage, and textbook outcome. The Cox regression model for multivariate analysis demonstrated that BMI, the ASA class, tumor size, pathological TNM stage, and textbook outcome were independent predictors of overall survival (Table 3). The prognostic factors for relapse-free survival in multivariate analysis were BMI, the ASA class, tumor size, pathological TNM stage, and textbook outcome (Table 4). Textbook outcome was an independent prognostic factor for both overall (hazard ratio [HR] 2.46, 95% confidence interval [CI] 1.28–4.73; *P* = 0.007) and relapse-free survival (HR 2.33, 95% CI 1.31–4.15, *P* = 0.004). The 3-year overall survival rate was significantly higher for patients who achieved textbook outcome than for those who did not (97.8% vs 87.2%; log-rank *P* = 0.012). Similarly, the 3-year relapse-free survival rate was significantly longer for patients who achieved textbook outcome than for those who did not (96.7% vs. 83.4%; log-rank *P* = 0.002). The overall and relapse-free survival features exhibited clear separation in the matched cohort; this demonstrated the prognostic effect of textbook outcome (Supplementary Fig. S2). However, the operative method was not an independent predictor of overall (*P* = 0.299) and relapse-free survival (*P* = 0.141). The HRs for overall and relapse-free survival in the RTG group compared with those in the LTG group were 0.60 (95% CI 0.23–1.57) and 0.52 (95% CI 0.21–1.25), respectively. In the matched cohort, age, pathological TNM stage, and textbook outcome were independent prognostic factors for overall and relapse-free survival. The operation method was not associated with overall and relapse-free survival, even in the matched cohort (Supplementary Tables S5, S6).

**Table 3:** Univariate and multivariate Cox regression models for overall survival.

Factors	Frequency (n = 395)	Univariate model		Multivariate model	
		HR (95%CI)	*P*	HR (95%CI)	*P*
Age, year^a^	395	1.63 (1.25–2.23)	** < 0.001**	1.24 (0.94–1.64)	0.136
**Sex**					
Male	265	1			
Female	130	0.84 (0.45–1.56)	0.574		
**Body mass index, kg/m**^**2**^					
< 23	151	1		1	
≥ 23	244	0.46 (0.26–0.81)	**0.008**	0.48 (0.26–0.89)	**0.019**
**ASA**					
1	201	1		1	
2	184	2.18 (1.16–4.09)	**0.016**	1.69 (0.81–3.53)	0.163
3	10	5.75 (2.09–15.84)	**0.001**	6.02 (1.86–19.40)	**0.003**
**Tumor histology**					
Differentiated	234	1			
Undifferentiated	161	0.75 (0.40–1.40)	0.364		
**Tumor location**					
Upper third	272	1			
Middle to lower third	123	1.32 (0.74–2.37)	0.347		
Tumor size, cm	395	1.29 (1.20–1.39)	** < 0.001**	1.16 (1.04–1.28)	**0.006**
**Operation method**					
Laparoscopy	321	1		1	
Robot	74	0.45 (0.18–1.14)	0.091	0.60 (0.23–1.57)	0.299
Operation time, min^b^	395	1.04 (0.99–1.10)	0.119		
Estimated blood loss, ml^c^	395	1.02 (1.01–1.03)	**0.003**	1.001 (0.99–1.02)	0.882
**Pathological TNM stage**^**d**^					
I	257	1		1	
II	67	1.72 (0.68–4.34)	0.252	1.94 (0.73–5.11)	0.182
III	71	7.75 (4.14–14.50)	** < 0.001**	4.21 (1.86–9.53)	**0.001**
**Textbook outcome**					
Achieved	243	1		1	
Failed to achieve	152	2.18 (1.23–3.87)	**0.008**	2.46 (1.28–4.73)	**0.007**

Statistically significant values (*P* < 0.05) are given in bold.

*HR* hazard ratio, *CI* confidence interval, *ASA* American Society of Anaesthesiologists.

^a^The hazard ratio shown is for every 10 year increase in age.

^b^The hazard ratio shown is for every 10 min increase in operation time.

^c^The hazard ratio shown is for every 10 ml increase in estimated blood loss.

**Table 4:** Univariate and multivariate Cox regression models for relapse-free survival.

Factors	Frequency (n = 395)	Univariate model		Multivariate model	
		HR (95% CI)	*P*	HR (95% CI)	*P*
Age, year^a^	395	1.65 (1.30–2.09)	** < 0.001**	1.21 (0.94–1.57)	0.146
**Sex**					
Male	265	1			
Female	130	0.80 (0.45–1.40)	0.427		
**Body mass index, kg/m**^**2**^					
< 23	151	1		1	
≥ 23	244	0.51 (0.30–0.84)	**0.009**	0.56 (0.33–0.96)	**0.034**
**ASA**					
1	201	1		1	
2	184	1.86 (1.08–3.22)	**0.026**	1.43 (0.75–2.71)	0.277
3	10	4.25 (1.60–11.28)	**0.004**	5.43 (1.77–16.69)	**0.003**
**Tumor histology**					
Differentiated	234	1			
Undifferentiated	151	0.73 (0.42–1.27)	0.267		
**Tumor location**					
Upper third	272	1			
Middle to lower third	123	1.03 (0.60–1.76)	0.921		
Tumor size, cm	395	1.30 (1.21–1.39)	** < 0.001**	1.11 (1.01–1.23)	**0.027**
**Operation method**					
Laparoscopy	321	1		1	
Robot	74	0.43 (0.19–1.00)	0.051	0.52 (0.21–1.25)	0.141
Operation time, min^b^	395	1.04 (0.99–1.09)	0.120		
Estimated blood loss, ml^c^	395	1.02 (1.003–1.03)	**0.012**	1.00 (0.98–1.01)	0.669
**Pathological TNM stage**^**d**^					
I	257	1		1	
II	67	2.14 (0.93–4.93)	0.075	2.30 (0.95–5.56)	0.064
III	71	10.72 (5.98–19.21)	** < 0.001**	7.08 (3.39–14.82)	**0.004**
**Textbook outcome**					
Achieved	243	1		1	
Failed to achieve	152	2.03 (1.22–3.38)	**0.007**	2.33 (1.31–4.15)	**0.004**

Statistically significant values (*P* < 0.05) are given in bold.

*HR* hazard ratio, *CI* confidence interval, *ASA* American Society of Anaesthesiologists.

^a^The hazard ratio shown is for every 10 year increase in age.

^b^The hazard ratio shown is for every 10 min increase in operation time.

^c^The hazard ratio shown is for every 10 ml increase in estimated blood loss.

^d^Stage, according to the 8th edition of the American Joint Committee on Cancer staging system for gastric cancer.

## Discussion

We performed propensity score matched analysis to adjust for potential selection bias, and short- and long-term outcomes were similar between the groups following successful matching. The RTG group showed similar operative outcomes compared with the LTG group, except for a longer operative time. Textbook outcome as a composite measure was comparably achieved in both groups. Regarding the long-term outcomes of both groups, similar overall and relapse-free survival rates were observed. Textbook outcome was an independent predictor for survival; however, the operative method was not an independent prognostic factor of survival.

To date, most studies on short-term outcomes following minimally invasive total gastrectomy focused on single factors, such as bleeding, retrieved lymph nodes, hospital stay, complications, and mortality, to measure surgical quality^23^. Unlike standard surgical quality measures, textbook outcome integrates metrics for oncologically sound surgical treatment, and metrics for measuring clinical events (void of complications and prolonged admissions). Thus, textbook outcome is a helpful measure in evaluating the quality of gastrectomy^9^. To the best of our knowledge, this study is the first to evaluate the short-term outcomes of RTG compared with those of LTG for patients with gastric cancer using textbook outcome.

In this study, textbook outcome was comparable between the RTG and LTG groups. Because robotic and laparoscopic surgeries fall into the same domain of minimally invasive approaches, it may be a major obstacle for robotic surgery to offer significant surgical advantages compared to laparoscopic surgery. Moreover, because RTG has been used for proximal gastric cancer since 2009 in our institution, RTG included initial and early experiences, while LTG included experiences after the learning curve in this study. Therefore, it would be more difficult to show the difference between the two methods in terms of textbook outcome. Nevertheless, RTG appeared to be non-inferior to LTG in our patient cohort, and comparisons after completion of the learning curve in both groups could lead to improved results or even superiority of the robotic approach. However, the number of cases after completion of the learning curve in the RTG group might be too small to reach an effective conclusion.

Cost effectiveness of robotic surgery is a concern for surgeons. High cost of the equipment and its maintenance seems to be a major drawback of robotic surgery. There is also the additional cost to training a surgeon, although the number of cases needed to overcome the learning curve of robotic surgery is less than that of laparoscopic surgery. In addition to training cost, the limited number of robotic systems in each institution may reduce surgical training opportunities for robotic surgery. Therefore, surgeon might not be able to take full advantage of robotic system if surgeon is not yet acquainted with it. In Korea, since the cost of robotic gastrectomy is not covered by National Health Insurance yet, patients have to bear separate fees. However, recently, private health insurance companies have begun to partially cover the costs of robotic surgery in Korea. Thus, the cost burden of robotic surgery may reduce for patients with private health insurance. In addition, the increased number of robotic surgeries can minimize depreciation and maintenance costs. If the medical expenses associated with robotic surgery decrease in the future, high cost will not be an absolute disadvantage of robotic gastrectomy.

It might be difficult to ascertain whether robotic gastrectomy could have overwhelming benefits for short-term outcomes, since laparoscopic gastrectomy is already a well-established and satisfactorily safe procedure. Nevertheless, robotic gastrectomy has several apparent advantages of compared to laparoscopic gastrectomy, which contribute to reducing invasiveness and surgical trauma. Articulated devices afford surgeons to perform each surgical procedure even more accurately and precisely. Although articulated devices have also become available for laparoscopic surgery, they are still limited and require technical improvement. Other apparent advantages include suppression of hand tremor, which is effective in maintaining a stable surgical field and in preventing organ injury. These enhanced technologies can help surgeons to perform operations comfortably. In the present study, unintended adjacent organ injury or resection, and conversion to open surgery were not observed as intraoperative complications in the RTG group, which may support these advantages.

Some studies have also demonstrated that robotic gastrectomy has the advantage of lesser blood loss, higher number of retrieved lymph nodes, shorter hospital stay, and shorter learning curve than those of laparoscopic gastrectomy^14,24,25^. The number of retrieved lymph nodes and duration of hospital stay were included in the metrics of textbook outcome, while blood loss was not included in the quality metrics. In this study, blood loss tended to be lower in the RTG group than in the LTG group, although this was not statistically significant. Since intraoperative bleeding during gastrectomy is associated with the risk of tumor recurrence^26–28^, modifying the textbook outcome to include the metric for intraoperative blood loss may yield more valuable results in gastric cancer surgery.

In proximal gastric cancer, total gastrectomy with the minimally invasive approach including digestive tract reconstruction and splenic hilar lymphadenectomy is technically feasible; however, it is still a challenging procedure for many surgeons^29–31^. In addition, compared with distal gastrectomy, total gastrectomy inevitably causes some complications and is also a risk factor for postoperative morbidity and mortality^32^. Likewise, in this study, the overall complication rate was 31.1% in both groups of the matched cohort, which was also higher than the complication rate of distal gastrectomy in previous studies^3,5^. Further, the severe complication metric showed the most negative effect on the textbook outcome in both groups, while the other eight quality metrics were over 90% in both groups. Although minimally invasive total gastrectomy reduced postoperative complications compared with open total gastrectomy^33–35^, a way to minimize complications after minimally invasive total gastrectomy should be considered.

Textbook outcome was achieved in 71.9% (284/395) of the study cohort, which was higher than the 32.1% reported in the Dutch Upper Gastrointestinal Cancer Audit group study^6^. The quality metric of “at least 15 lymph nodes examined” had the greatest negative effect on the textbook outcome in the Dutch study, while the severe complication metric showed the most negative effect on the textbook outcome in this study. The exclusion of palliative gastrectomy for survival analysis, relatively younger age of the patients, low ASA class, and earlier cancer stage of the study cohort may have contributed to the higher achievement of the textbook outcome. As such, differences in patient characteristics and tumor biology would result in different achievement rates of each quality metric between this study and the Dutch study^36,37^.

In this study, overall and relapse-free survival rates were similar between both groups. In multivariable analysis, the operative method was not a prognostic factor for survival, whereas achieving textbook outcome was a prognostic factor for survival. Western studies also demonstrated that textbook outcome is strongly associated with long-term survival in gastric cancer surgery^8,9^. Therefore, the achievement of textbook outcome was significantly related to the improvement of survival rate, suggesting that the quality of surgery plays an important role in the survival of gastric cancer patients regardless of the operative method.

This study has the following limitations. First, this study had a small-scale, single-center retrospective design. Therefore, we cannot exclude potential selection bias by the surgeon, although clinicopathological characteristics of the patients were well balanced between the groups after matching; of note, more advanced tumors were generally treated with laparoscopic surgery. Second, the generalizability of our findings to the Western population is uncertain because this Eastern study cohort had relatively early gastric cancer with a small number of events. Third, there were only six patients with recurrence and/or death in the RTG group; this was a relatively small number. Before any conclusive statements regarding prognostic factors for survival in this patient group can be made, large-scale randomized clinical trials are required to increase the power of the statistical analysis and validate our results. Finally, we did not evaluate functional long-term outcomes between the two groups. Thus, we could not compare the quality of life after minimally invasive total gastrectomy.

In conclusion, textbook outcome and 3-year survival after RTG were similar to those after LTG for gastric cancer. Although a longer operation time and higher costs remain major concerns, RTG might be considered a feasible and safe treatment option for gastric cancer. Achieving textbook outcome is strongly associated with improved long-term survival in patients undergoing total gastrectomy for gastric cancer.

## Supplementary Information

Supplementary Information.
