# Supplementary material for: Textbook outcome and survival of robotic versus laparoscopic total gastrectomy for gastric cancer: a propensity score matched cohort study
Source: Sci Rep. 2021 Jul 28;11:15394. doi: 10.1038/s41598-021-95017-3 (PMC8319437; doi:10.1038/s41598-021-95017-3)
Supplement: Supplementary file 1 — Supplementary Information. [file 41598_2021_95017_MOESM1_ESM.docx]

**Supplementary Online Contents**

**Textbook outcome and survival of robotic versus laparoscopic total gastrectomy for gastric cancer: A propensity score matched cohort study**

Chul Kyu Roh, MD^1,2^, Soomin Lee, MD^1,2^, Sang-Yong Son, MD^1,2^, Hoon Hur, MD, PhD^1,2^, Sang-Uk Han, MD, PhD^1,2^

^1^Department of Surgery, Ajou University School of Medicine, Suwon, Republic of Korea

^2^Gastric Cancer Center, Ajou University Medical Center, Suwon, Republic of Korea

**Corresponding author and address for reprint requests:**

Sang-Uk Han, MD, PhD

Department of Surgery, Ajou University School of Medicine

164, World cup-ro, Yeongtong-gu, Suwon-si, Gyunggi-do 16499, Republic of Korea

Tel: 82-31-219-5200; Fax: 82-31-3-219-5755; E-mail: hansu@ajou.ac.kr

**Contents**

1. eFigure 1. Patient flow diagram

2. eFiguire 2. Kaplan-Meier three-year overall and relapse-free curves according to the achievement of textbook outcome in the matched cohort

3. eTable 1. Distribution of early and late complications before and after propensity score matching

4. eTable 2. Textbook outcome and each quality metric before and after propensity score matching

5. eTable 3. Details of intraoperative complications

6. eTable 4. Recurrence patterns before and after propensity score matching

7. eTable 5. Univariate and multivariate Cox regression models for overall survival in the matched cohort

8. eTable 6. Univariate and multivariate Cox regression models for relapse-free survival in the matched cohort

**eFigure 1. Patient flow diagram**

**
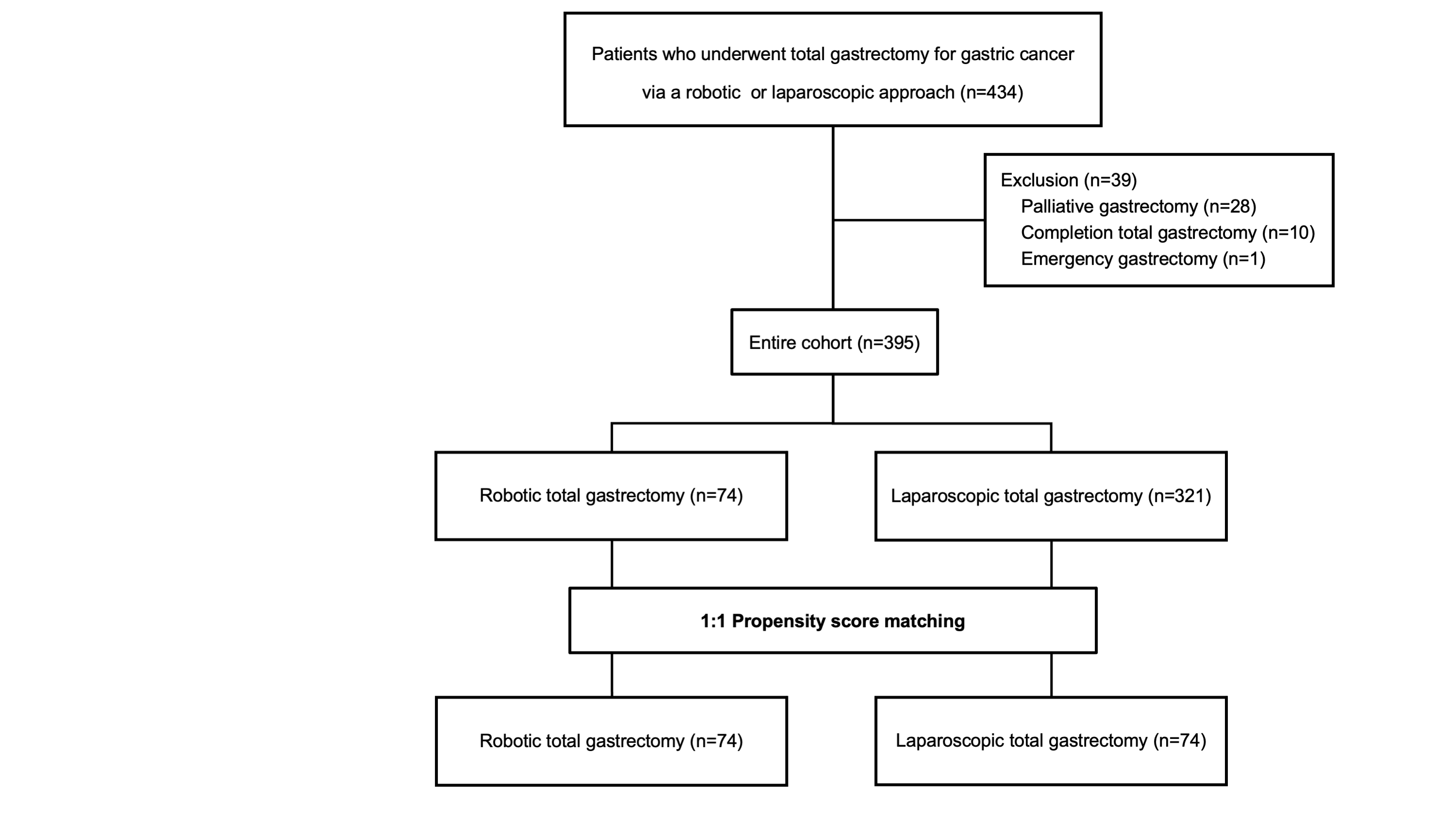
**

**eFiguire 2. Kaplan–Meier curves for 3-year overall and relapse-free survival according to the achievement of textbook outcome in the matched cohort**

~~
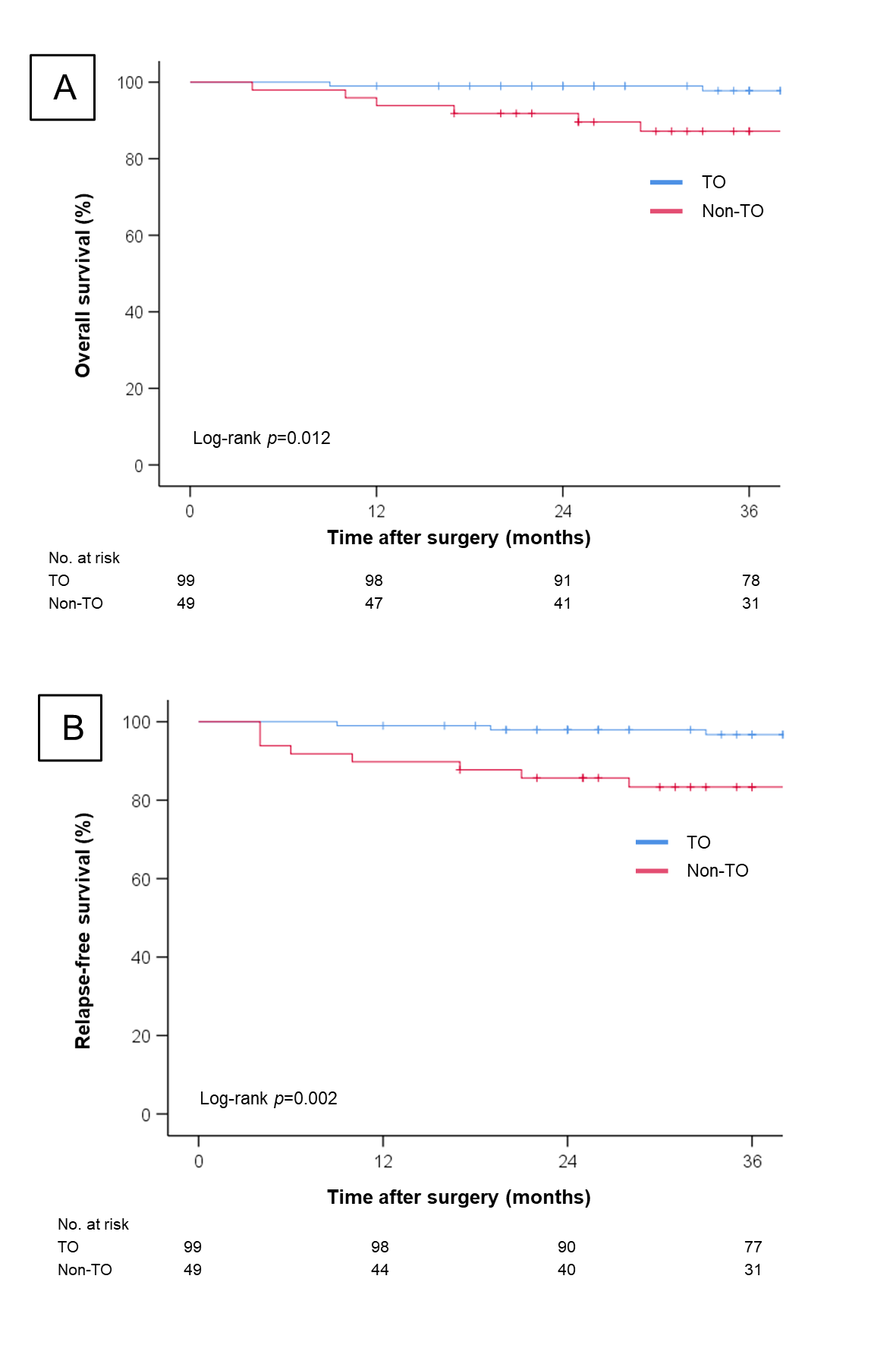
~~

TO : achieved textbook outcome; Non-TO : failure to achieve textbook outcome

**eTable 1. Distribution of early and late complications before and after propensity score matching**

| **Variables** | **Entire cohort** | |  | **Matched cohort** | |  |
| --- | --- | --- | --- | --- | --- | --- |
|  | **Robot**  (n=74) | **Laparoscopy**  (n=321) | ***P*** | **Robot** (n=74) | **Laparoscopy** (n=74) | ***P*** |
| Total **early complication**-subject experience | 23 (31.1) | 80 (24.9) | 0.304 | 23 (31.1) | 23 (31.1) | >0.999 |
| **Local complication** | 17 (23.0) | 54 (16.8) | 0.240 | 17 (23.0) | 20 (27.0) | 0.705 |
| Intra-abdominal fluid collection | 1 (1.4) | 4 (1.2) |  | 1 (1.4) | 1 (1.4) |  |
| Intra-abdominal bleeding | 1 (1.4) | 6 (1.9) |  | 1 (1.4) | 1 (1.4) |  |
| Intra-luminal bleeding | 1 (1.4) | 5 (1.6) |  | 1 (1.4) | 2 (2.7) |  |
| Anastomotic leakage | 3 (4.1) | 7 (2.2) |  | 3 (4.1) | 5 (6.8) |  |
| Anastomotic stricture | 2 (2.7) | 2 (0.6) |  | 2 (2.7) | 1 (1.4) |  |
| Ileus | 5 (6.8) | 15 (4.7) |  | 5 (6.8) | 6 (8.1) |  |
| Pancreatitis/Pancreatic fistula | 2 (2.7) | 4 (1.2) |  | 2 (2.7) | 1 (1.4) |  |
| Wound | 2 (2.7) | 11 (3.4) |  | 2 (2.7) | 3 (4.1) |  |
| **Systemic complication** | 4 (5.4) | 18 (5.6) | >0.999 | 4 (5.4) | 2 (2.7) | 0.681 |
| Pulmonary | 3 (4.1) | 12 (3.7) |  | 3 (4.1) | 1 (1.4) |  |
| Urinary | 0 (0.0) | 2 (0.6) |  | 0 (0.0) | 1 (1.4) |  |
| Hepatic | 1 (1.4) | 3 (0.9) |  | 1 (1.4) | 0 (0.0) |  |
| Cardiac | 0 (0.0) | 1 (0.3) |  | 0 (0.0) | 0 (0.0) |  |
| Other complications | 2 (2.7) | 8 (2.5) | >0.999 | 2 (2.7) | 1 (1.4) | >0.999 |
| Total **late complication**-subject experience | 9 (12.2) | 33 (10.3) | 0.676 | 9 (12.2) | 7 (9.5) | 0.792 |
| Anastomotic stricture | 5 (6.8) | 16 (5.0) |  | 5 (6.8) | 5 (6.8) |  |
| Intestinal obstruction | 4 (5.4) | 10 (3.1) |  | 4 (5.4) | 1 (1.4) |  |
| Post-gastrectomy syndrome | 0 (0.0) | 5 (1.6) |  | 0 (0.0) | 1 (1.4) |  |
| Others | 0 (0.0) | 2 (0.6) |  | 0 (0.0) | 0 (0.0) |  |

Data are expressed as n (%) unless otherwise specified.

**eTable 2. Textbook outcome and each quality metric before and after propensity score matching**

|  | **Entire cohort** | | | **Matched cohort** | | |
| --- | --- | --- | --- | --- | --- | --- |
|  | **Robot** (n=74) | **Laparoscopy** (n=321) | ***P*** | **Robot** (n=74) | **Laparoscopy** (n=74) | ***P*** |
| No intraoperative complication | 72 (97.3) | 311 (96.9) | >0.999 | 72 (97.3) | 73 (98.6) | >0.999 |
| Negative resection margin | 74 (100.0) | 319 (99.4) | >0.999 | 74 (100.0) | 74 (100.0) | NA |
| Retrieved lymph nodes >15 | 73 (98.6) | 316 (98.4) | >0.999 | 73 (98.6) | 74 (100.0) | >0.999 |
| No severe complication (≥grade II) | 57 (77.0) | 261 (81.3) | 0.417 | 57 (77.0) | 57 (77.0) | >0.999 |
| No reintervention | 66 (89.2) | 290 (90.3) | 0.829 | 66 (89.2) | 63 (85.1) | 0.624 |
| No readmission to ICU | 72 (97.3) | 314 (97.8) | 0.678 | 72 (97.3) | 71 (95.9) | >0.999 |
| Length of hospital stay ≤21 days | 70 (94.6) | 314 (97.8) | 0.131 | 70 (94.6) | 70 (94.6) | >0.999 |
| No mortality | 74 (100.0) | 74 (100.0) | NA | 74 (100.0) | 74 (100.0) | NA |
| No readmission 30 days after discharge | 68 (91.9) | 295 (91.9) | >0.999 | 68 (91.9) | 70 (94.6) | 0.745 |
| Textbook outcome | 52 (70.3) | 232 (72.3) | 0.775 | 52 (70.3) | 56 (75.7) | 0.579 |

Data are expressed as n (%) unless otherwise specified.

ICU, intensive care unit; NA, not applicable

**eTable 3. Details of intraoperative complications**

|  | **Entire cohort** | | |
| --- | --- | --- | --- |
|  | **Robot** (n=74) | **Laparoscopy** (n=321) | ***P*** |
| No intraoperative complication | 72 (97.3) | 311 (96.9) | >0.999 |
| Intraoperative complications | 2 (2.7) | 10 (3.1) |  |
| Conversion to open surgery for any reason | 0 | 4 |  |
| Intraoperative transfusion due to intraoperative blood loss | 2 | 4 |  |
| Splenectomy due to splenic artery injury | 0 | 1 |  |
| Diaphragm injury | 0 | 1 |  |

Data are expressed as n (%) unless otherwise specified.

**eTable 4. Recurrence patterns before and after propensity score matching**

|  | **Entire cohort** | |  | **Matched cohort** | |  |
| --- | --- | --- | --- | --- | --- | --- |
|  | **Robot** (n=74) | **Laparoscopy** (n=321) | ***P*** | **Robot** (n=74) | **Laparoscopy** (n=74) | ***P*** |
| Total patients with recurrence | 2 (2.7) | 31 (9.7) | 0.061 | 2 (2.7) | 5 (6.8) | 0.442 |
| Hematogenous | 1 (1.4) | 6 (1.9) |  | 1 (1.4) | 1 (1.4) |  |
| Peritoneal | 1 (1.4) | 19 (5.9) |  | 1 (1.4) | 4 (5.4) |  |
| Distant lymph node | 0 (0.0) | 1 (0.3) |  | 0 (0.0) | 0 (0.0) |  |
| Locoregional | 0 (0.0) | 0 (0.0) |  | 0 (0.0) | 0 (0.0) |  |
| Mixed | 0 (0.0) | 5 (1.6) |  | 0 (0.0) | 0 (0.0) |  |

Data are expressed as n (%) unless otherwise specified.

**eTable 5. Univariate and multivariate Cox regression models for overall survival in the matched cohort**

|  |  | **Univariate model** | | **Multivariate model** | |
| --- | --- | --- | --- | --- | --- |
| Factors | **Frequency** (n=148) | **HR (95%CI)** | ***P*** | **HR (95%CI)** | ***P*** |
| **Age**, year ^a^ | 148 | 2.67 (1.66-4.28) | **<0.001** | 2.15 (1.35-3.44) | **0.001** |
| **Sex** |  |  |  |  |  |
| Male | 84 | 1 |  |  |  |
| Female | 64 | 0.43 (0.14-1.34) | 0.145 |  |  |
| **Body mass index,** kg/m^2^ |  |  |  |  |  |
| <23 | 65 | 1 |  |  |  |
| ≥23 | 83 | 1.13 (0.40-3.16) | 0.824 |  |  |
| **ASA** |  |  |  |  |  |
| 1 | 101 | 1 |  |  |  |
| 2 | 44 | 2.35 (0.83-6.71) | 0.109 |  |  |
| 3 | 3 | 5.22 (0.64-42.48) | 0.122 |  |  |
| **Tumor histology** |  |  |  |  |  |
| Differentiated | 77 | 1 |  |  |  |
| Undifferentiated | 71 | 0.83 (0.30-2.34) | 0.724 |  |  |
| **Tumor location** |  |  |  |  |  |
| Upper third | 98 | 1 |  |  |  |
| Middle to lower third | 50 | 0.89 (0.30-2.61) | 0.834 |  |  |
| **Tumor size,** cm | 148 | 1.28 (1.08-1.51) | **0.005** | 1.06 (0.86-1.31) | 0.568 |
| **Operation method** |  |  |  |  |  |
| Laparoscopy | 74 | 1 |  | 1 |  |
| Robot | 74 | 0.46 (0.16-1.34) | 0.155 | 0.37 (0.12-1.16) | 0.089 |
| **Operation time**, min ^b^ | 148 | 1.04 (0.95-1.13) | 0.438 |  |  |
| **Estimated blood loss**, ml ^c^ | 148 | 1.03 (1.002-1.05) | **0.032** | 1.04 (0.99-1.08) | 0.090 |
| **Pathological TNM stage** ^d^ |  |  |  |  |  |
| I | 97 | 1 |  | 1 |  |
| II | 33 | 2.19 (0.52-9.22) | 0.283 | 6.93 (1.12-42.68) | **0.037** |
| III | 18 | 9.67 (3.06-30.59) | **<0.001** | 14.41 (2.95-70.46) | **0.001** |
| **Textbook outcome** |  |  |  |  |  |
| Achieved | 99 | 1 |  | 1 |  |
| Failed to achieve | 49 | 3.44 (1.22-9.69) | **0.019** | 4.23 (1.21-14.75) | **0.024** |

^a^ the hazard ratio shown is for every 10 year increase in age.

^b^ the hazard ratio shown is for every 10 min increase in operation time.

^c^ the hazard ratio shown is for every 10 ml increase in estimated blood loss.

^d^ Stage, according to the 8th edition of the American Joint Committee on Cancer staging system for gastric cancer.

HR, hazard ratio; CI, confidence interval; ASA, American Society of Anaesthesiologists.

**eTable 6. Univariate and multivariate Cox regression models for relapse-free survival in the matched cohort**

|  |  | **Univariate model** | | **Multivariate model** | |
| --- | --- | --- | --- | --- | --- |
| Factors | **Frequency** (n=148) | **HR (95%CI)** | ***P*** | **HR (95%CI)** | ***P*** |
| **Age**, year ^a^ | 148 | 2.56 (1.66-3.95) | **<0.001** | 1.90 (1.23-2.94) | **0.004** |
| **Sex** |  |  |  |  |  |
| Male | 84 | 1 |  | 1 |  |
| Female | 64 | 0.37 (0.12-1.13) | 0.082 | 0.62 (0.17-2.23) | 0.462 |
| **Body mass index,** kg/m^2^ |  |  |  |  |  |
| <23 | 65 | 1 |  |  |  |
| ≥23 | 83 | 1.13 (0.40-3.16) | 0.824 |  |  |
| **ASA** |  |  |  |  |  |
| 1 | 101 | 1 |  |  |  |
| 2 | 44 | 1.83 (0.68-4.91) | 0.231 |  |  |
| 3 | 3 | 4.08 (0.52-32.23) | 0.183 |  |  |
| **Tumor histology** |  |  |  |  |  |
| Differentiated | 77 | 1 |  |  |  |
| Undifferentiated | 71 | 0.84 (0.32-2.21) | 0.726 |  |  |
| **Tumor location** |  |  |  |  |  |
| Upper third | 98 | 1 |  |  |  |
| Middle to lower third | 50 | 0.75 (0.26-2.13) | 0.585 |  |  |
| **Tumor size,** cm | 148 | 1.27 (1.08-1.50) | **0.005** | 1.09 (0.89-1.34) | 0.418 |
| **Operation method** |  |  |  |  |  |
| Laparoscopy | 74 | 1 |  | 1 |  |
| Robot | 74 | 0.46 (0.16-1.34) | 0.155 | 0.39 (0.13-1.15) | 0.089 |
| **Operation time**, min ^b^ | 148 | 1.03 (0.95-1.12) | 0.435 |  |  |
| **Estimated blood loss**, ml ^c^ | 148 | 1.02 (1.002-1.05) | **0.036** | 1.03 (0.99-1.06) | 0.155 |
| **Pathological TNM stage** ^d^ |  |  |  |  |  |
| I | 97 | 1 |  | 1 |  |
| II | 33 | 1.77 (0.44-7.11) | 0.420 | 4.17 (0.77-22.55) | 0.097 |
| III | 18 | 9.43 (3.26-27.28) | **<0.001** | 11.67 (2.78-49.05) | **0.001** |
| **Textbook outcome** |  |  |  |  |  |
| Achieved | 99 | 1 |  | 1 |  |
| Failed to achieve | 49 | 4.26 (1.54-11.26) | **0.005** | 4.24 (1.28-14.11) | **0.018** |

^a^ the hazard ratio shown is for every 10 year increase in age.

^b^ the hazard ratio shown is for every 10 min increase in operation time.

^c^ the hazard ratio shown is for every 10 ml increase in estimated blood loss.

^d^ Stage, according to the 8th edition of the American Joint Committee on Cancer staging system for gastric cancer.

HR, hazard ratio; CI, confidence interval; ASA, American Society of Anaesthesiologists.
